# Sparse Blind Deconvolution with Nonconvex Optimization for Ultrasonic NDT Application

**DOI:** 10.3390/s20236946

**Published:** 2020-12-04

**Authors:** Xuyang Gao, Yibing Shi, Kai Du, Qi Zhu, Wei Zhang

**Affiliations:** 1 School of Automation Engineering, University of Electronic Science and Technology of China, No.2006, Xiyuan Avenue, West Hi-tech Zone, Chengdu 611731, China; xuyanggao@std.uestc.edu.cn (X.G.); ybshi@uestc.edu.cn (Y.S.); 202022060402@std.uestc.edu.cn (K.D.); 2 College of Mechatronic Engineering, Southwest Petroleum University, No.8, Xindu Road, Xindu District, Chengdu 610500, China; 201231010016@swpu.edu.cn

**Keywords:** ultrasonic detection, sparse blind deconvolution, nonconvex optimization, blind gain calibration

## Abstract

In the field of ultrasonic nondestructive testing (NDT), robust and accurate detection of defects is a challenging task because of the attenuation and noising of the ultrasonic wave from the structure. For determining the reflection characteristics representing the position and amplitude of ultrasonic detection signals, sparse blind deconvolution methods have been implemented to separate overlapping echoes when the ultrasonic transducer impulse response is unknown. This letter introduces the $\ell_{1}/\ell_{2}$ ratio regularization function to model the deconvolution as a nonconvex optimization problem. The initialization influences the accuracy of estimation and, for this purpose, the alternating direction method of multipliers (ADMM) combined with blind gain calibration is used to find the initial approximation to the real solution, given multiple observations in a joint sparsity case. The proximal alternating linearized minimization (PALM) algorithm is embedded in the iterate solution, in which the majorize-minimize (MM) approach accelerates convergence. Compared with conventional blind deconvolution algorithms, the proposed methods demonstrate the robustness and capability of separating overlapping echoes in the context of synthetic experiments.

## 1. Introduction

Ultrasonic nondestructive testing (NDT) is an established detection technology, which can accurately characterize multilayer or composite materials [1]. The primary ultrasonic NDT tocology mostly considered in practical systems is the pulse-echo method, which evaluates the surface and defects based on the estimation of the time of arrival (TOA), time of flight (TOF), time of flight diffraction (TOFD) and the time difference of arrival (TDOA) of received signals [2]. However, extracting the implicit information is difficult with the distortion and attenuation of the captured signals under noisy environments. Hence, the inspection aims to correctly estimate the parameter values, or separate superimposed waveforms, representing the physical properties.

The conventional method to improve the detection resolution is to increase the transducer frequency, leading to a decrease in acoustic penetration [3]. The other accepted practice estimates the waveform parameters by the Gaussian echo model (or other modified models) and prior information from the transducer [4]. These approaches have some limitations, such as reliance on hardware design and finite propagation distortion. Generally, the signal sample of ultrasonic pulse-echo testing is regarded as the convolution result between the reflection function over the propagation path and the system pulse response. Therefore, deconvolution signal processing methods can be implemented to improve temporal resolution, which significantly affects revealing any small defect object masked by superimposed echoes. The existing mainstream methods are divided into the parametric method and optimization theory method.

Parametric methods depend on prior knowledge of waveform and design specific structures to obtain the estimated parameters. With the assumption that the reflection sequence was sparse, Chang [5] applied the Gauss-Newton (GN) algorithm to parameter estimation of the basic waveform and employed the split variable augmented Lagrangian shrinkage algorithm (SALSA) in the reflection sequence solution. Jin [6] determined the reference echo signal via maximum likelihood estimation (MLE) and achieved sparse deconvolution results by the orthogonal matching pursuit (OMP) algorithm. Bossmann [7] deconvolved the superimposed echo signal by removing the additive noise with a particular matching pursuit (MP) algorithm. The variational mode decomposition (VMD) algorithm was applied to deconvolute the signal by reducing the noise level, which increased the resolution of several defects in different positions [8]. Another class of methods is the minimum entropy deconvolution (MED) technique without acquiring prior assumptions, and the main idea is to find the inverse filter to make the output as sparse as possible [9,10]. In the research conducted by Li [11], $\ell_{0}$ norm transformation is applied to enhance the sparsity of MED outputs and eliminate the iteration overflow caused by the long inverse filter. Morphological filtering with the sparse MED algorithm was proposed in [12], where the scale of the signal characteristics can be adaptively selected according to morphological filtering results. Under the premise of mastering the relevant information about the instrument, these algorithms were efficient [3]. Moreover, transforming the ultrasonic parameter estimation into sparse deconvolution was proved to be potentially possible.

However, due to distortion of the waveform in the transmission process, the received signal has a high probability that only the morphological hypothesis (e.g., the sparse prior) can satisfy. Hence, many researchers in recent years have directly abstracted ultrasonic pulse-echo deconvolution into nonconvex optimization with sparsity assumptions, where both the transmitted wavelet and the reflectivity function are unknown. Li [13] constructed a reliable two-step computing framework and applied phase lifting to initialize the blur function. Under statistical assumptions of the signal, Qu [14] studied the multichannel sparse blind deconvolution (MCS-BD) problem via the gradient descent (GD) algorithm. When multiple input signals are present, finding sparse vectors in a subspace can be directly converted into blind calibration [15]. In order to improve the stability of initialization, $\ell_{2}$ norm projection [16], least-squares (LS) [17], and pruned tree search [18] are proposed. Zhang [19] explored the influence of symmetric structure on the iterative solution and recovered the shift truncation over the kernel sphere. In simulation experiments, methods based on optimization theory have better antinoise performance than parametric methods.

This letter mainly focuses on sparse blind deconvolution for pulse-echo application, which is an essential branch of ultrasonic NDT. The current challenges of solving this problem with optimization theory are as follows:

- Although accurate estimation parameters can be obtained through finite iterations, utilizing the optimization framework is time-consuming [20].
- Without prior waveform information, the optimization process is nonconvex, which means that a robust initialization algorithm is inevitable.

We construct a two-step algorithm based on the nonconvex optimization inspired by [13] to solve these problems. The nonconvex model for multichannel ultrasonic signal deconvolution using the $\ell_{1}/\ell_{2}$ ratio is proposed, the solution process of which is divided into two parts. First, with multiple observations, the convolution process is transformed into a sparse blind correction problem in the sparsity case, and the initial estimations of system response and sparse sequence are obtained by the alternating direction method of multipliers (ADMM) [21]. The proximal alternating linearized minimization (PALM) algorithm [22] is applied to solve the nonconvex and nonsmooth optimization problems over each individual received sample. The majorize-minimize (MM) approximation is introduced with symmetric positive definite (SPD) matrix compensation [23] for convergence accelerating purposes. The performance of the proposed algorithms is investigated through simulation analysis and numerical experiment, which demonstrates that the superimposed signals can be significantly faster deconvoluted with reasonable initial estimations.

The remainder of this letter is organized as follows. Section 2 models the multiple blind deconvolution problem and introduces the initialization method and iteration algorithm. Section 3 evaluates the proposed method with performance analysis and compares it with classical deconvolution methods. Section 4 concludes and summarizes this paper.

## 2. Optimization Models and Methods

### 2.1. Convolution Model of Ultrasonic Inspection

**Figure 1:**
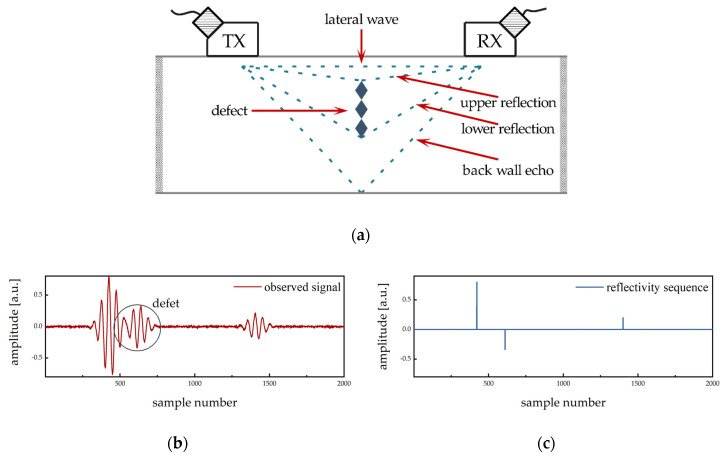
Diagram for time of flight diffraction (TOFD) testing and example of A-scan signal: (**a**) TOFD arrangement for defect inspection; (**b**) observed receive signal sample by convolution; (**c**) hypothetical reflectivity sequence.

The ultrasonic excitation signal is usually generated by a high-voltage pulse generator, which stimulates a sensor made of piezoelectric material. The generated wave propagates in the measured object and forms a superimposed ultrasonic echo after reflection by defects and surfaces. We integrate into one signal the measurement model from TOFD and the A-scan experiments regarding the specific ultrasonic pulse-echo application, as illustrated in Figure 1.

To analyze the inspection and achieve separation in the time domain, one can regularly assume that the received signal convolves results by the generated ultrasonic pulse-echo with the reflectivity sequence. The latter is a small combination of the reflection coefficients. Hence, the convolution model of the multiple measurement process can be represented as

(1) $$y_{l}={\bar{h}}_{l}*{\bar{x}}_{l}\;+w_{l},$$

where ∗ denotes the convolution operation, 1≤l≤L, $y_{l}\in {\mathbb{R}}^{n}$ is the observation sample at the receiving end, ${\bar{h}}_{l}\in {\mathbb{R}}^{s}$ represents the impulse response (or the blurring function), ${\bar{x}}_{l}\in {\mathbb{R}}^{n}$ is the ith reflected sequence with the prior sparsity and $w_{l}$ is the additive noise. We utilized the Gaussian echo model to analyze the impulse response and assumed that the relevant parameters were stable over time in a complete measurement process. Thus:

(2) $${\bar{h}}_{l}:=h\left(\theta ,\;t\right)=\beta e^{-\alpha t^{2}}\cos\left(2\pi \omega t+\phi \right),$$

with the parameter vector θ=(β, α, ω, ϕ), which describes amplitude, bandwidth factor, center frequency and phase, respectively. In this letter, we are only interested in the waveform of h(θ, t) without solving the specific θ. Hence by ignoring the subscript l in Equation (1), we propose the following blind deconvolution model to recover h and x from y:

(3) F(x, h)=f(x, h)+g(x, h),

where

(4) $$f\left(x,\;h\right)=f_{0}\left(x,\;h\right)\;+\lambda \frac{\left\|x_{1}\right\|}{\left\|x_{2}\right\|}$$

is the least-squares objective function $f_{0}\left(x,\;h\right)=\frac{1}{2}\|h*x-y{\|}_{2}^{2}$ with $\ell_{1}/\ell_{2}$ the ratio regularization function. λ is a regularization parameter. g(x, h) contains prior information about the optimization objects, such as amplitude range, and is the continuous convex function in the domain. The $\ell_{1}/\ell_{2}$ function is the normalized version of the $\ell_{1}$ function and holds a scale-invariant, which behaves correctly for deconvolution with irregular blurring functions [24] by considering the following nonconvex optimization algorithm:

(5) $$\left(\hat{x},\;\hat{h}\right):=\underset{h,\;x}{\mathrm{argmin}}F\left(x,\;h\right),\;\;\;\;\mathrm{for}\;\mathrm{all}\;1\;\leq \;l\;\leq L.$$

Obviously, the solution to this problem is not uniquely identifiable, and we can always find a solution from the equivalence class $y_{l}=\left(\epsilon R_{l}^{+}h\right)*\left(\epsilon^{-1}R_{l}^{-}{\bar{x}}_{l}\right)$, ϵ≠0 without noise, where R is the shift symmetry matrix [13]. The convergence to the correct solution relies on the initial estimation $\left(x_{0},\;h_{0}\right)$ of Equation (5). When the center frequency parameter is small, or the basic waveform is the centered Gaussian filter, the global optimal solution can be obtained by random initialization or wavelet initialization function [16]. Because of the constraint from the regularization function, the initialization of the reflected sequence is relatively relaxed. For avoiding the computational difficulty and local optimal solution of solving the model, a well-produced initialization $h_{0}$ is crucial to the success of Equation (5).

### 2.2. Initialization Based on Blind Gain Calibration

Without considering the noise, we modify Equation (1) as:

(6) $$y_{l}=C\left(g\right){\bar{x}}_{l},$$

where C(g) is the Toeplitz circulant matrix of $g={\left[h_{1},\;h_{2},\ldots ,\;h_{s},0,0,\ldots ,0\right]}^{T}\in {\mathbb{R}}^{n}$, and can be written as

(7) $$C\left(g\right)=\left[\begin{array}{ccc} g_{1} & \cdots & g_{2}\\ \vdots & \ddots & \vdots \\ g_{n} & \cdots & g_{1} \end{array}\right].$$

By denoting that multiple measurements can be written in matrix form, i.e., $Y=\left[y_{2},\;y_{2},\;\ldots ,y_{L}\right]\;\in \;{\mathbb{R}}^{n\times L}$ and $X=\left[{\bar{x}}_{1},\;{\bar{x}}_{2},\;\ldots ,{\bar{x}}_{L}\right]\;\in \;{\mathbb{R}}^{n\times L}$, we rewrite Equation (6) as:

(8) Y=C(g)X.

The circulant matrix C(g) has the equivalent eigenvalue decomposition, thus:

(9) $$C\left(g\right):=F^{H}\mathrm{diag}\left(\overset{˘}{g}\right)F,$$

where F is the n×n unitary discrete Fourier transform (DFT) matrix, and $\overset{˘}{g}=\sqrt{n}Fg\in {\mathbb{C}}^{n}$. By applying the DFT matrix F to both sides of Equation (8), we have:

(10) $$FY=\mathrm{diag}\left(\overset{˘}{g}\right)FX.$$

Let $\overset{˘}{Y}=FY$ and, the measurement process can be parameterizing in a linear representation:

(11) $$\tau_{i}{\overset{˘}{y}}_{i,l}=F_{i}^{H}{\bar{x}}_{l},\tau_{i}:=\frac{1}{{\overset{˘}{g}}_{i}},\;\mathrm{for}\;\mathrm{all}\;1\;\leq \;i\;\leq n\;\mathrm{and}\;1\;\leq \;l\;\leq L,$$

in which ${\overset{˘}{y}}_{i,l}$ is the element of the ith row and the lth column in $\overset{˘}{Y}$, $F_{i}^{H}$ denotes the ith row of F. The bilinear inverse problem of $\left(\overset{˘}{g},\;X\right)$ is transformed into a linear inverse problem of (τ, X). Even in the case of actual noise interference [25], we also can solve the problem:

(12) $$\mathrm{diag}\left(\tau \right)\overset{˘}{Y}\approx FX.$$

The proposed initialization algorithm becomes equivalent to blind gain calibration proposed in [26], which leads to solving:

(13) $$\left(\hat{\tau },\;\hat{X}\right):=\underset{\tau ,\;\;X}{\mathrm{argmin}\;}\|X{\|}_{1},\;\;s.t.\;\mathrm{diag}\left(\tau \right)\overset{˘}{Y}=FX.$$

Evidently, it satisfies for the pair (0, 0). To avoid this, we introduce an additional linear constraint on τ, thus:

(14) $$1^{T}\tau =c,$$

where $1^{T}=\left[1,\;1,\ldots ,\;1\right]$. The combination of Equations (13) and (14) is a convex problem, which can be solved using the existing solvers.

However, numerical simulations indicate that the direct use of this linear equality constraint complicates the problem. Therefore, we use the ADMM algorithm, which updates local subproblems to coordinate the global problem and solve Equation (13). An equivalent operator of the sum constraint is applied in the iteration process to simplify the initialization process.

First, the augmented Lagrangian function of Equation (13) with the expansion of Equation (11) is expressed as:

(15) $${ℒ}_{\rho }\left(X,\tau ,\lambda \right)=\sum_{l=1}^{L}\|{\bar{x}}_{l}{\|}_{1}+\sum_{i=1}^{n}\sum_{l=1}^{L}\lambda_{i,l}\left(F_{i}^{H}{\bar{x}}_{l}-\tau_{i}{\overset{˘}{y}}_{i,l}\right)+\frac{\rho }{2}\sum_{i=1}^{n}\sum_{l=1}^{L}{\left(F_{i}^{H}{\bar{x}}_{l}-\tau_{i}{\overset{˘}{y}}_{i,l}\right)}^{2},$$

where ρ>0 is the penalty parameter and λ is the Lagrange multiplier matrix. The Equation (13) processes in following steps by updating parameters individually:

(16) $$\{\begin{array}{c} X^{\left(k+1\right)}:=\underset{X}{\mathrm{argmin}}\;{ℒ}_{\rho }\left(X,\;\tau^{\left(k\right)},\lambda^{\left(k\right)}\right)\\ \tau^{\left(k+1\right)}:=\underset{\tau }{\mathrm{argmin}}\;{ℒ}_{\rho }\left(X^{\left(k+1\right)},\tau ,\lambda^{\left(k\right)}\right)\\ \lambda^{\left(k+1\right)}:=\lambda^{\left(k\right)}+\rho \left(FX^{\left(k+1\right)}-\mathrm{diag}\left(\tau^{\left(k+1\right)}\right)\overset{˘}{Y}\right) \end{array}.$$

For update for X, $\tau^{\left(k\right)}$ can be regarded as constant values, and the minimization problem is reduced to:

(17) $$\underset{X}{\mathrm{argmin}}\;{ℒ}_{\rho }\left(X,\tau^{\left(k\right)},\lambda^{\left(k\right)}\right)≔\;\underset{X}{\mathrm{argmin}}\;\sum_{l=1}^{L}\|{\bar{x}}_{l}{\|}_{1}+\sum_{l=1}^{L}\lambda_{l}^{T}\left(F{\bar{x}}_{l}-{\overset{˘}{b}}_{l}^{\left(k\right)}\right)+\frac{\rho }{2}\sum_{l=1}^{L}\|F{\bar{x}}_{l}-{\overset{˘}{b}}_{l}^{\left(k\right)}{\|}_{2}^{2},$$

where ${\overset{˘}{b}}_{l}^{\left(k\right)}$ is the lth column in $\mathrm{diag}\left(\tau^{\left(k+1\right)}\right)\overset{˘}{Y}$. The optimization problem for ${\bar{x}}_{l}$ is stated as follows:

(18) $$\underset{{\bar{x}}_{l}}{\mathrm{argmin}}\;\|{\bar{x}}_{l}{\|}_{1}+\lambda_{l}^{T}\left(F{\bar{x}}_{l}-{\overset{˘}{b}}_{l}^{\left(k\right)}\right)+\frac{\rho }{2}\|F{\bar{x}}_{l}-{\overset{˘}{b}}_{l}^{\left(k\right)}{\|}_{2}^{2}.$$

Let $\zeta_{0}\left(x\right)=\lambda_{l}^{T}\left(Fx-{\overset{˘}{b}}_{l}^{\left(k\right)}\right)+\frac{\rho }{2}\|Fx-{\overset{˘}{b}}_{l}^{\left(k\right)}{\|}_{2}^{2}$, and thus:

(19) $$\|\nabla \zeta_{0}\left(x^{\prime }\right)-\nabla \zeta_{0}{\left(x\right)\|}_{2}=\rho \|F^{T}F{\left(x^{\prime }-x\right)\|}_{2}\leq \rho \|F^{T}{F\|}_{2}\|{\left(x^{\prime }-x\right)\|}_{2}=\rho \|{F\|}_{2}^{2}\|{\left(x^{\prime }-x\right)\|}_{2},$$

which means an ℒ-Lipschitzian gradient for every $x^{\prime }\in \;{\mathbb{R}}^{n}$ (see Appendix A). The Lipschitzian constants ${ℒ}_{0}$ of $\zeta_{0}\left(x\right)$ is $\rho {\left\|F\right\|}_{2}^{2}$. Hence, the update rule of ${\hat{x}}_{l}$ is defined based on an approximation:

(20) $${\bar{x}}_{l}^{\left(k+1\right)}:=\underset{{\bar{x}}_{l}}{\mathrm{argmin}}\;\|{\bar{x}}_{l}{\|}_{1}+\;\frac{{ℒ}_{0}}{2}\;\|{\bar{x}}_{l}-z^{\left(k\right)}\|{}_{2}^{2},$$

where:

(21) $$z^{\left(k\right)}={\bar{x}}_{l}^{\left(k\right)}-\frac{1}{{ℒ}_{0}}\nabla \zeta_{0}\left({\bar{x}}_{l}^{\left(k\right)}\right)$$

Because $\|{\bar{x}}_{l}{\|}_{1}$ is the $\ell_{1}$ norm function, the iterative shrinkage-thresholding (IST) method [27] can be used to find a closed-form solution:

(22) $${\bar{x}}_{l|j}^{\left(k+1\right)}=\underset{u}{\mathrm{argmin}}\;\frac{{\left(u-z_{j}^{\left(k\right)}\right)}^{2}}{2}+\frac{\left|u\right|}{{ℒ}_{0}}=\mathrm{soft}\left(z_{j}^{\left(k\right)},\;\frac{1}{{ℒ}_{0}}\right),$$

where ${\bar{x}}_{l|j}^{\left(k+1\right)}$ means the jth element of ${\bar{x}}_{l}^{\left(k\right)}$ (resp. $z_{j}^{\left(k\right)}$). soft(·) is the soft-thresholding function, which is defined as:

(23) soft(u,v)=sign(u)max[|u|−v,0].

Mote that $X^{\left(k+1\right)}$ is separable and consists of ${\bar{x}}_{l|j}^{\left(k+1\right)}$, the update of $X^{\left(k+1\right)}$ in Equation (13) can be rewritten as:

(24) $$X^{\left(k+1\right)}:=\mathrm{soft}(X^{\left(k\right)}-\;\frac{1}{{ℒ}_{0}}F^{T}\left(\lambda^{\left(k\right)}+\;\rho \left(FX^{\left(k\right)}-\mathrm{diag}\left(\tau^{\left(k\right)}\right)\overset{˘}{Y}\right),\;\frac{1}{{ℒ}_{0}}\right).$$

For updating $\tau^{\left(k\right)}$, X is regarded as constant values, and thus:

(25) $$\tau_{i}^{\left(k+1\right)}:=\;\underset{\tau_{i}}{\mathrm{argmin}}\;-\overset{L}{\underset{l=1}{\sum }}\lambda_{i,l}\tau_{i}{\overset{˘}{y}}_{i,l}+\frac{\rho }{2}\overset{L}{\underset{l=1}{\sum }}{\left(F_{i}^{H}{\bar{x}}_{l}^{\left(k+1\right)}-\tau_{i}{\overset{˘}{y}}_{i,l}\right)}^{2}=\underset{\tau_{i}}{\mathrm{argmin}}\;{ℒ}_{\rho }^{\prime }\left(X^{\left(k+1\right)},\;\tau ,\lambda^{\left(k\right)}\right),$$

and when $\nabla {ℒ}_{\rho }^{\prime }\left(X^{\left(k+1\right)},\;\tau_{i},\lambda^{\left(k\right)}\right)=0$, $\tau_{i}^{\left(k+1\right)}$ is represented as:

(26) $$\tau_{i}^{\left(k+1\right)}:=\frac{\sum_{l=1}^{l}\lambda_{i,l}{\overset{˘}{y}}_{i,l}+\rho \sum_{l=1}^{l}F_{i}^{H}{\bar{x}}_{l}^{\left(k+1\right)}{\overset{˘}{y}}_{i,l}}{\rho \sum_{l=1}^{l}{\overset{˘}{y}}_{i,l}^{2}}=\frac{U_{i}^{\left(k+1\right)}}{V_{i}},$$

where $U,V\in {\mathbb{C}}^{n}$, and:

(27) $$U^{\left(k+1\right)}=\mathrm{diag}\left((\lambda^{\left(k\right)}+\rho M_{i,l}\right){\overset{˘}{Y}}^{T}),\;\;V=\mathrm{diag}\left(\rho \overset{˘}{Y}{\overset{˘}{Y}}^{T}\right),\;\;M=FX^{\left(k+1\right)}.$$

Similar to $X^{\left(k+1\right)}$, we use $U^{\left(k+1\right)}/V$ directly to update $\tau^{\left(k+1\right)}$. Considering the constraints from formula Equation (14), we modify the update method of $X^{\left(k+1\right)}$ as:

(28) $$\tau_{i}^{\left(k+1\right)}:=\frac{U_{i}^{\left(k+1\right)}+\delta^{\left(k+1\right)}}{V_{i}},\;\;\;\;\delta^{\left(k+1\right)}=\frac{c-\sum_{i=i}^{n}\frac{U^{\left(k+1\right)}}{\;V_{l}}}{\sum_{i=i}^{n}\frac{1}{\;V_{l}}},$$

where $\mathrm{trace}\left(\mathrm{diag}\left(\tau^{\left(k+1\right)}\right)\right)=c$ satisfies the sum constraint.

Thus, through Equations (16), (24), and (28), the impulse response and sparse sequences from each measurement are estimated up to specific scaling. Despite the arbitrary selection of c and the assumption without noise, the initialized pair $\left(x_{0},\;h_{0}\right)$ is close enough to the ground truth, and guarantees Equation (5) can converge to the global optimal solution approximately.

### 2.3. Alternating Optimization Method Based on PALM

The basic PALM method is proposed for minimization of a sum of finite functions and requires the smooth function (or partially Lipschitz properties). However, the $\ell_{1}/\ell_{2}$ ratio is both nonconvex and nonsmooth, which means the smoothed $\ell_{1}/\ell_{2}$ ratio proposed in [16] can replace the $\ell_{1}/\ell_{2}$ ratio in f(x,h). Hence, we rewrite Equation (4) as $f\left(x,h\right)=f_{0}\left(x,h\right)+\phi \left(x\right)$, and:

(29) $$\phi \left(x\right)=\lambda \log\left(\frac{\ell_{1,\alpha }\left(x\right)}{\ell_{2,\eta }\left(x\right)}\right),\;\;\;\;\ell_{1,\alpha }\left(x\right)=\overset{n}{\underset{i=1}{\sum }}\sqrt{x_{i}^{2}+\alpha^{2}}-\alpha ,\;\;\;\;\ell_{2,\eta }\left(x\right)=\sqrt{\overset{n}{\underset{i=1}{\sum }}x_{i}^{2}+\eta^{2}},$$

which can be smooth approximations for sparse representation. Besides, we assume that g(x,h) is separable and is a proper, lower semicontinuous, convex function. Thus:

(30) $$g\left(x,h\right)=g_{1}\left(x\right)+g_{2}\left(h\right),$$

where $g_{1}$ and $g_{2}$ are the indicator functions with prior knowledge. Equation (5) is represented as:

(31) $$\underset{h,x}{\mathrm{argmin}}\;F\left(x,h\right):=f_{0}\left(x,h\right)+\phi \left(x\right)+g_{1}\left(x\right)+g_{2}\left(h\right).$$

By starting with the initial estimates $\left(x^{\left(0\right)},\;h^{\left(0\right)}\right)$ obtained from the initialization, we generate the sequence via the scheme:

(32) $$\{\begin{array}{c} x^{\left(k+1\right)}≔\underset{x}{\mathrm{argmin}}\;F\left(x,h^{\left(k\right)}\right)\\ h^{\left(k+1\right)}≔\underset{h}{\mathrm{argmin}}\;F\left(x^{\left(k+1\right)},h\right) \end{array}.$$

The PALM for solution of Equation (32) are defined respectively by:

(33) $$\{\begin{array}{c} x^{\left(k+1\right)}≔{\mathrm{prox}}_{g_{1},c^{\left(k\right)}}\left(x^{\left(k\right)}-\frac{1}{c^{\left(k\right)}}\nabla_{x}\;f\left(x^{\left(k\right)},\;h^{\left(k\right)}\right)\right)\\ h^{\left(k+1\right)}≔{\mathrm{prox}}_{g_{2},d^{\left(k\right)}}\left(h^{\left(k\right)}-\frac{1}{d^{\left(k\right)}}\nabla_{h}\;f\left(x^{\left(k+1\right)},\;h^{\left(k\right)}\right)\right) \end{array},$$

where the proximal operator is defined by the proximal map, which is associated to a specific function, i.e.,

(34) $${\mathrm{prox}}_{\sigma ,t}\left(z\right)=\underset{x}{\mathrm{argmin}}\;\sigma \left(x\right)+\frac{t}{2}\|x-{z\|}_{2}^{2}.$$

$c^{\left(k\right)}$ and $d^{\left(k\right)}$ are the partial Lipschitz constants of ∇ f (see Appendix A).

The solution of the problem is converted to determine the appropriate $c^{\left(k\right)}$ and $d^{\left(k\right)}$, so that the function Equation (33) converges. From idea of gradient descent, $c^{\left(k\right)}$ and $d^{\left(k\right)}$ can be selected as:

(35) $$c^{\left(k\right)}=\gamma_{1}{ℒ}_{1}\left(h^{\left(k\right)}\right),\;\;\;\;d^{\left(k\right)}=\gamma_{2}{ℒ}_{2}\left(x^{\left(k+1\right)}\right),\;\;\;\;\gamma_{1},\gamma_{2}>0,$$

where ${ℒ}_{1}$ and ${ℒ}_{2}$ are the Lipschitz constants for $\nabla_{x}\;f$ and $\nabla_{h}\;f$ [22]. Similar to the algorithm in [28], $x^{\left(k+1\right)}$ can be considered constants when updating $h^{\left(k+1\right)}$, and:

(36) $${ℒ}_{2}\left(x^{\left(k+1\right)}\right)\approx {\left\|(T_{x^{\left(k+1\right)}}\right)}^{T}T_{x^{\left(k+1\right)}}{\|}_{2},$$

where $T_{x}:H^{n}\to H^{n\times n}$ is the Toeplitz matrix operator. For $x^{\left(k+1\right)}$, we rewrite the update step as:

(37) $$x^{\left(k+1\right)}≔{\mathrm{prox}}_{g_{1},\gamma_{1}^{-1}A{\left(x^{\left(k\right)},h^{\left(k\right)}\right)}^{-1}}\left({\tilde{x}}^{\left(k\right)}\right),\;\;\;\;\;\;\;\;\;\;\;\;\;\;\;\;\;\;\;{\tilde{x}}^{\left(k\right)}=x^{\left(k\right)}-\frac{1}{\gamma_{1}}A{\left(x^{\left(k\right)},\;h^{\left(k\right)}\right)}^{-1}\nabla_{x}\;f\left(x^{\left(k\right)},h^{\left(k\right)}\right),$$

and $A\left(x^{\left(k\right)},h^{\left(k\right)}\right)$ is the symmetric positive definite (SPD) matrix which satisfies the MM principle [23], which can be obtained by building majorizing approximations for f(x,h) [29]. Thus:

(38) $$A\left(x^{\left(k\right)},h^{\left(k\right)}\right)=\left({ℒ}_{1}^{\prime }+\frac{9\lambda }{8\eta^{2}}\right)I_{n}+{\mathrm{diag}}_{\ell_{1,\alpha }\left(x\right)}\left(x^{\left(k\right)}\right),\;\;\;\;\;\;\;\;\;\;\;\;\;\;\;\;\;\;\;{\;\mathrm{diag}}_{\ell_{1,\alpha }\left(x\right)}\left(x\right)=\mathrm{diag}\left({\left[{\left(x_{i}^{2}+\alpha^{2}\right)}^{-\frac{1}{2}}\right]}_{1\leq i\leq n}\right),$$

where ${ℒ}_{1}^{\prime }$ is the Lipschitzian constants of $\nabla_{x}f_{0}\left(x,h^{\left(k\right)}\right)$. By Equation (8), we have:

(39) $$\nabla_{x}f_{0}\left(x,h^{\left(k\right)}\right)=M^{T}\left(Mx-y\right),\;\;\;\;M=F^{H}\mathrm{diag}\left(Fh^{\left(k\right)}\right)F,$$

and ${ℒ}_{1}^{\prime }={\left\|M\right\|}_{2}^{2}$. The other items in $A\left(x^{\left(k\right)},h^{\left(k\right)}\right)$ are given by the proposition established in [16].

Thus far, for each measurement sample $y_{i}$, we can obtain the corresponding estimated $\left({\hat{x}}_{i},{\hat{h}}_{i}\right)$ by solving Equation (32) with a global initialization $\left(X_{o},\;h_{0}\right)$ from Equation (16).

## 3. Simulation Results

### 3.1. Stable Initialization with Phase Transitions

**Figure 2:**
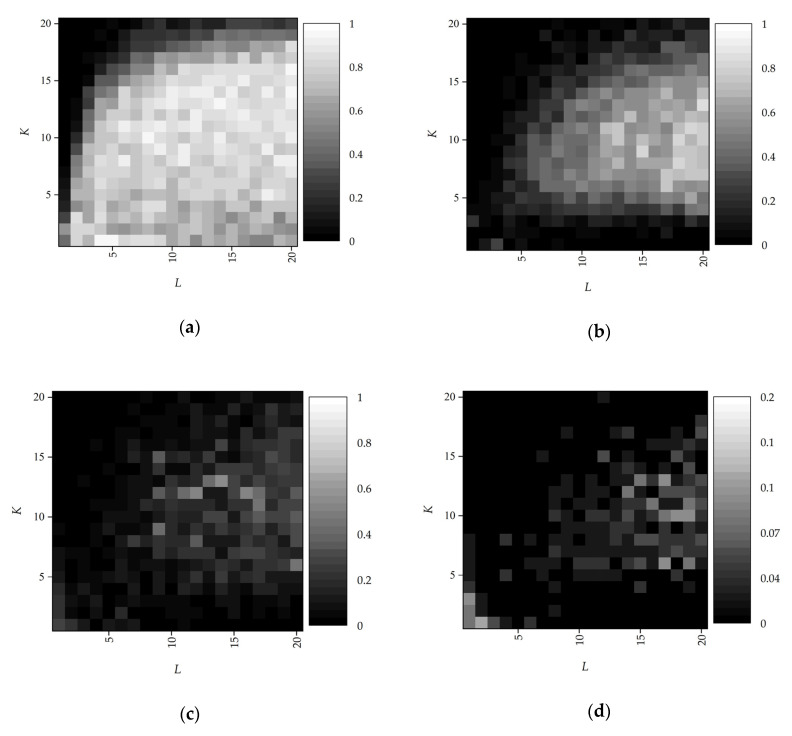
Phase transitions of the proposed initialization algorithm with different noise levels: (**a**) noise free; (**b**) σ=0.01; (**c**) σ=0.02; (**d**) σ=0.03. The gray bar denotes the success rate.

Phase diagrams, which demonstrate the empirical probability of success over a range of sparsity and samples for a fixed sampling window length, are implemented to evaluate the feasibility of the proposed initialization algorithm. We classify a success initialization $h_{0}$ of h if $\|h_{0}-{h\|}_{2}/{\left\|h\right\|}_{2}\leq 0.3$. Figure 2 presents variations of the initialization success rate by phase transitions, concerning the K-sparsity of each ${\bar{x}}_{l}$ and the number of samples L. The additive noise $w_{l}$ is considered as white Gaussian noise with the variance $\sigma^{2}$. The length of the sequence n=100 and the recovery success rate are calculated using 50 Monte Carlo simulations on every grid point. We rescale the estimated $h_{0}$ to have the same norm as the correct h.

As shown in Figure 2a, the initialization algorithm can estimate h correctly when the sparsity and the number of samples are appropriate. To achieve successful initialization with Equation (16), L must scale linearly with K. However, when K does not satisfy the sparse hypothesis, i.e., K≪n does not hold, blind gain calibration cannot achieve convergence to the neighborhood of h. In the case of noise interference, the proposed method has a high probability of obtaining $h_{0}$ with increased samples. Because the influence of noise is not introduced in Equation (11), few samples lead to additional errors for the iterative recovery. Meanwhile, as shown in Figure 2d, the probability of successful initialization decreases with serious noise interference.

### 3.2. Numerical Comparison for Deconvolution Evaluation

**Figure 3:**
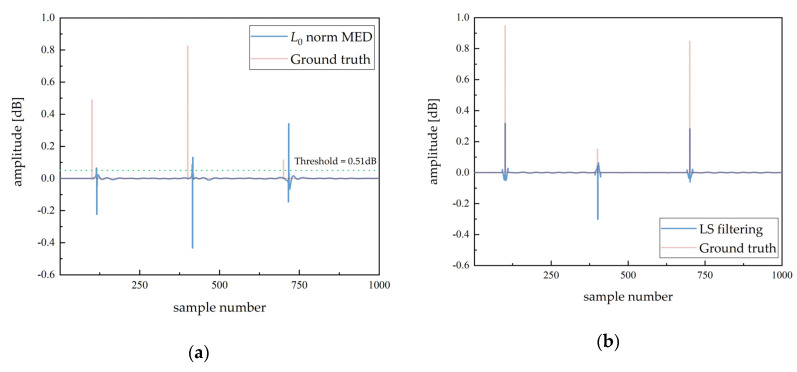
Comparison of parametric methods: (**a**) $\ell_{0}$ norm MED (the threshold is used to determine detection points); (**b**) LS algorithm.

Figure 3 illustrates the comparison of conventional parametric deconvolution methods numerically with the same noise variance (σ=0.02). The regularization parameters and indicator functions of [12,29] are adjusted in this comparison. In the case of K=3, L=20, and n=1000, offset and polarity reversal presents in the conventional fast deconvolution methods, such as MED [11] and LS [17]. The MED algorithm uses an inverse filter to solve sparse reflection sequence directly. However, the uncorrelation between the inverse filter and the blurring function results in deviation and fluctuation, as shown in Figure 3a. Besides, the MED algorithm extracts the arrival time by manually modulating the threshold, which reduces detection stability. The LS algorithm estimates the blurring function and analyzes the concentration of the error, where the relatively accurate time parameters can be obtained. The regularized coefficient of noise is based on the prior information of the signal. Therefore, the deviation appears in the estimation of amplitude parameters. The reflection sequence, estimated by LS (Figure 2b), is sparser than that estimated by MED. Although MED uses sparse $\ell_{0}$ constraint, the noise also affects the deconvolution results.

**Figure 4:**
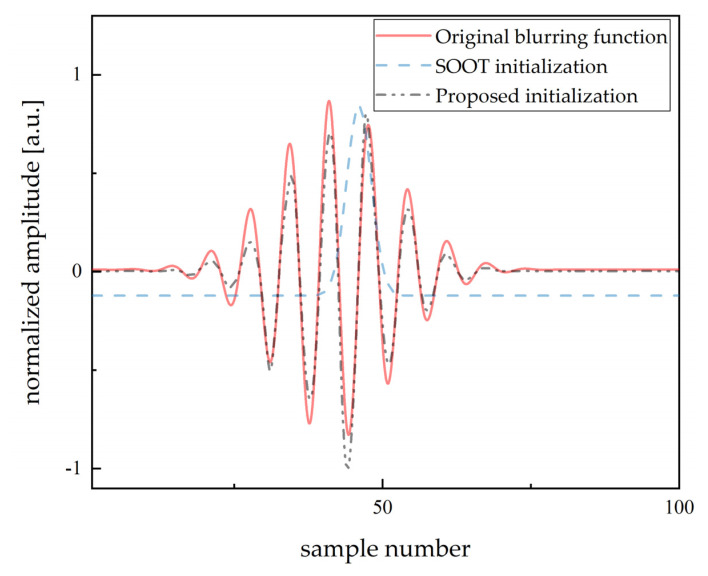
Comparison of initialization algorithms.

Compared with parametric methods, the method based on optimization theory has better adaptability to the superimposed echo. Therefore, to evaluate the performance of the proposed algorithm, the sparsity is increased appropriately. Further, when K=20, L=20, and n=1000 (σ=0.02), Figure 4 shows the initialization comparison of the Smoothed One-Over-Two (SOOT) [29] algorithm and the proposed method. The centered Gaussian filter from SOOT is different from the basic ultrasonic waveform, where converging on local optimum is inevitable. Although the blind gain calibration is implemented with time cost, the proposed algorithm provides a starting point close to the original blurring function for alternating convergence.

**Figure 5:**
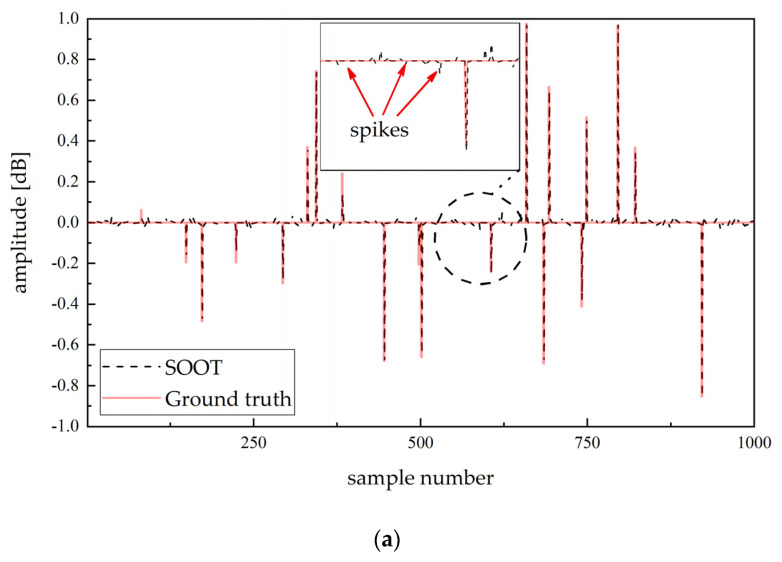

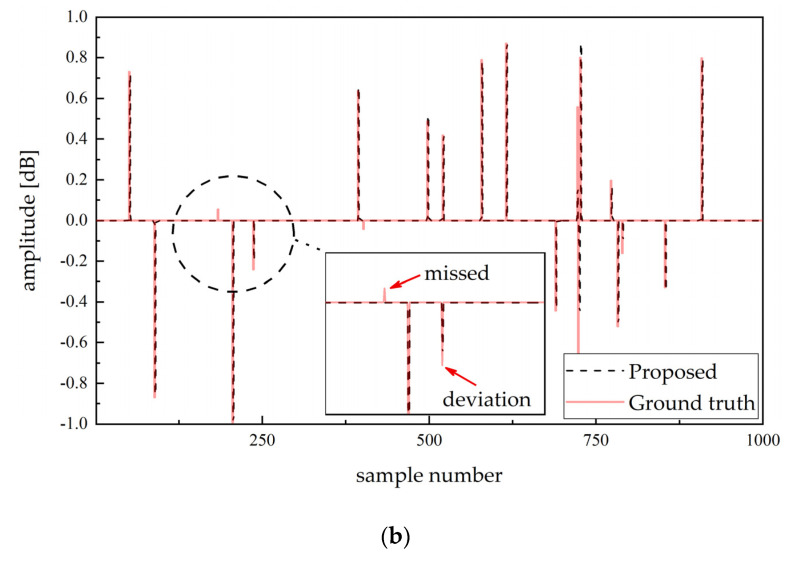
Deconvolution results of different methods with optimization theory: (**a**) SOOT; (**b**) proposed algorithm.

Figure 5 reveals the results of the initialization algorithm and alternating solution. Although the SOOT algorithm reduces the influence of initialization by variable metric strategies and increasing the number of subiterations, the spike interference still affects the solution where the initialization of the centered Gaussian filter is not applicable to the true ultrasonic response. Despite limited missing solutions and amplitude deviation, the proposed algorithm can accurately estimate the position and amplitude of the reflected sequence in the case of relative sparsity.

For a fair comparison, we used the same noise conditions and parameter settings as illustrated in [29], where the fixed noise standard variances (0.01, 0.02, and 0.03) and regularization parameters are considered. Table 1 compares the residual deconvolution error, where $\ell_{2,h}$ and $\ell_{1,x}$ represent the norm distances between the estimated values and the correct values for different noise levels, respectively. The running time is obtained by taking the average value from experiments, and the stopping criterion for iterative algorithms is set as $\|x^{\left(k+1\right)}-x^{\left(k\right)}{\|}_{1}\leq 10^{-3}$. Although the deconvolution based on LS [17] is superior in speed, the cost is a large estimation error. MED-based methods [9,10,11] achieve relatively good sparse sequence estimation in less computing time. However, because of the characteristics of the inverse filter, the basic response of the signal is inaccessible. A solution using alternate optimization can obtain a better solution at the cost of computing time. The CVX toolbox without improvement causes additional time consumption due to the introduction of random initialization. Besides, because the optimization conditions do not make specific constraints on the ultrasonic waveform, the estimation error of the blurring function is larger than that of the reflection sequence. Meanwhile, the proposed algorithm is significantly faster than the other algorithms based on nonconvex optimization. The proposed initialization is closer to the ground truth, where the algorithm can achieve the stopping criterion more quickly.

## 4. Conclusions

In this letter, a deconvolution method based on nonconvex optimization is proposed. By simplifying the problem to blind gain calibration, a two-step iterative initialization can effectively obtain the initial guesses for ultrasonic pulse-echo response and reflection sequence with the ADMM algorithm. Then, using $\ell_{1}/\ell_{2}$ ratio regularization, an exact algorithm based on the PALM framework is applied. Simulation and numerical analysis illustrate that the proposed method algorithm has the probability of successful initialization. In the algorithm comparison experiment, deconvolution results are obtained with high accuracy and acceptable time cost. 

The proposed method has potential gains in ultrasonic NDT applications for defect detection and sensor parameter estimation. When the specific instrument parameters are ambiguous or the propagation is distorted, the parameters representing the measured object can be obtained accurately through sparse blind deconvolution. However, the time cost limits the application of the algorithm in other areas (such as ultrasonic imaging) unless the processing speed is further improved.

## Figures and Tables

**Table 1:** Comparison of algorithm deconvolution results. The interpretation platform used the Intel Core i5-9400f processor with 16GB memory.

Algorithm	σ=0.01		σ=0.02		σ=0.03		Running Time (s)
	$\ell_{2,h}$	$\ell_{1,x}$	$\ell_{2,h}$	$\ell_{1,x}$	$\ell_{2,h}$	$\ell_{1,x}$	
LS	2.3981	1.1092	2.5495	1.5212	3.4781	2.5008	3.89
$\ell_{0}$ MED	\	0.8121	\	1.1002	\	1.3063	5.62
M-S-MED	\	0.7203	\	0.8212	\	1.0304	8.20
OMED	\	0.5548	\	0.6167	\	0.9033	10.34
$\ell_{1}/\ell_{2}$ CVX	1.5301	0.1023	1.6623	0.4180	2.0301	0.8902	65.29
SOOT	0.8923	0.0221	1.2340	0.0936	1.6721	0.1645	34.50
proposed	0.3622	0.0133	0.4528	0.0584	0.8973	0.1045	22.32

## Appendix A

For every k∈ℕ, the quadratic function defined as

(A1) $$\forall x\in {\mathbb{R}}^{N},\;\;\;\;Q\left(x,x_{k}\right)≔F\left(x_{k}\right)+{\left(x-x_{k}\right)}^{T}\nabla F\left(x_{k}\right)+\frac{1}{2}\|x-x_{k}{\|}_{A_{k}}^{2},$$

is a majorant function of $F\left(x_{k}\right)$. Thus:

(A2) $$\forall k\in \mathbb{N},\;\;\;\;v_{1}I_{N}≼A_{k}≼v_{2}I_{N},$$

for $v_{1},v_{2}\in [0,+\infty {]}^{2}$. If F is Lipschitz differentiable on dom R, yielding:

(A3) $$\forall x,y\in {\left(\mathrm{dom}\;R\right)}^{2},\;\;\;\;F\left(x\right)<F\left(y\right)+\left(x-y\right)\nabla F\left(x_{k}\right)+\frac{1}{2}\|x-{y\|}^{2},$$

for dom R is a convex set and $A_{k}=LI_{N}$, where L>0 is the Lipschitz constant of ∇F.

## References

[1] Katunin A., Wronkowicz-Katunin A., Dragan K. (2020). Impact Damage Evaluation in Composite Structures Based on Fusion of Results of Ultrasonic Testing and X-ray Computed Tomography. Sensors.

[2] Zhao N., Basarab A., Kouame D., Tourneret J.-Y. (2016). Joint Segmentation and Deconvolution of Ultrasound Images Using a Hierarchical Bayesian Model Based on Generalized Gaussian Priors. IEEE Trans. Image Process..

[3] Park Y., Choi A., Kim K. (2017). Monaural Sound Localization Based on Reflective Structure and Homomorphic Deconvolution. Sensors.

[4] Jeong H., Cho S., Zhang S., Li X. (2018). Acoustic nonlinearity parameter measurements in a pulse-echo setup with the stress-free reflection boundary. J. Acoust. Soc. Am..

[5] Chang Y., Zi Y., Zhao J., Yang Z., He W., Sun H. (2017). An adaptive sparse deconvolution method for distinguishing the overlapping echoes of ultrasonic guided waves for pipeline crack inspection. Meas. Sci. Technol..

[6] Jin H., Chen J., Yang K. (2016). A blind deconvolution method for attenuative materials based on asymmetrical Gaussian model. J. Acoust. Soc. Am..

[7] Bossmann F., Plonka G., Peter T., Nemitz O., Schmitte T. (2012). Sparse Deconvolution Methods for Ultrasonic NDT Application on TOFD and Wall Thickness Measurements. J. Nondestruct. Eval..

[8] Abdessalem B., Farid C. (2020). Resolution Improvement of Ultrasonic Signals Using Sparse Deconvolution and Variational Mode Decomposition Algorithms. Russ. J. Nondestruct. Test..

[9] Buzzoni M., Antoni J., D’Elia G. (2018). Blind deconvolution based on cyclostationarity maximization and its application to fault identification. J. Sound Vib..

[10] Cheng Y., Zhou N., Zhang W., Wang Z. (2018). Application of an improved minimum entropy deconvolution method for railway rolling element bearing fault diagnosis. J. Sound Vib..

[11] Li X., Li X., Liang W., Chen L. (2012). l(0)-norm regularized minimum entropy deconvolution for ultrasonic NDT & E. NDT E Int..

[12] Li M., Li X., Gao C., Song Y. (2019). Acoustic microscopy signal processing method for detecting near-surface defects in metal materials. NDT E Int..

[13] Li X., Ling S., Strohmer T., Wei K. (2019). Rapid, robust, and reliable blind deconvolution via nonconvex optimization. Appl. Comput. Harmon. Anal..

[14] Qu Q., Li X., Zhu Z. (2020). Exact Recovery of Multichannel Sparse Blind Deconvolution via Gradient Descent. SIAM J. Imaging Sci..

[15] Wang L., Chi Y. (2016). Blind Deconvolution From Multiple Sparse Inputs. IEEE Signal Process. Lett..

[16] Repetti A., Mai Quyen P., Duval L., Chouzenoux E., Pesquet J.-C. (2015). Euclid in a Taxicab: Sparse Blind Deconvolution with Smoothed l(1)/l(2) Regularization. IEEE Signal Process. Lett..

[17] Guan J., Wang X., Wang W., Huang L. (2017). Sparse Blind Speech Deconvolution with Dynamic Range Regularization and Indicator Function. Circuits Syst. Signal Process..

[18] Jing S., Hall J., Zheng Y.R., Xiao C. (2020). Signal Detection for Underwater IoT Devices With Long and Sparse Channels. IEEE Internet Things J..

[19] Zhang Y., Kuo H.-W., Wright J. (2020). Structured Local Optima in Sparse Blind Deconvolution. IEEE Trans. Inf. Theory.

[20] Yang H., Su X., Chen S. (2020). Blind Image Deconvolution Algorithm Based on Sparse Optimization with an Adaptive Blur Kernel Estimation. Appl. Sci..

[21] Boyd S., Parikh N., Chu E., Peleato B., Eckstein J. (2011). Distributed Optimization and Statistical Learning via the Alternating Direction Method of Multipliers. Found. Trends Mach. Learn..

[22] Bolte J., Sabach S., Teboulle M. (2014). Proximal alternating linearized minimization for nonconvex and nonsmooth problems. Math. Program..

[23] Chouzenoux E., Pesquet J.-C., Repetti A. (2014). Variable Metric Forward-Backward Algorithm for Minimizing the Sum of a Differentiable Function and a Convex Function. J. Opt. Theory Appl..

[24] Krishnan D., Tay T., Fergus R. Blind Deconvolution Using a Normalized Sparsity Measure. Proceedings of the 2011 IEEE Conference on Computer Vision and Pattern Recognition.

[25] Li Y., Lee K., Bresler Y. (2019). Blind Gain and Phase Calibration via Sparse Spectral Methods. IEEE Trans. Inf. Theory.

[26] Gribonval R., Chardon G., Daudet L. Blind calibration for compressed sensing by convex optimization. Proceedings of the 2012 IEEE International Conference on Acoustics, Speech and Signal Processing.

[27] Combettes P.L., Wajs V.R. (2005). Signal recovery by proximal forward-backward splitting. Multiscale Model. Simul..

[28] Sun Y., Babu P., Palomar D.P. (2017). Majorization-Minimization Algorithms in Signal Processing, Communications, and Machine Learning. IEEE Trans. Signal Process..

[29] Mai Quyen P., Oudompheng B., Nicolas B., Mars J.I. Sparse deconvolution for moving-source localization. Proceedings of the 2016 IEEE International Conference on Acoustics, Speech and Signal Processing Proceedings.

